# Supplementary material for: Genome assembly of wisent (Bison bonasus) uncovers a deletion that likely inactivates the THRSP gene
Source: Commun Biol. 2024 Nov 27;7:1580. doi: 10.1038/s42003-024-07295-y (PMC11603333; doi:10.1038/s42003-024-07295-y)
Supplement: Supplementary file 2 — Supplementary Information [file 42003_2024_7295_MOESM2_ESM.pdf]

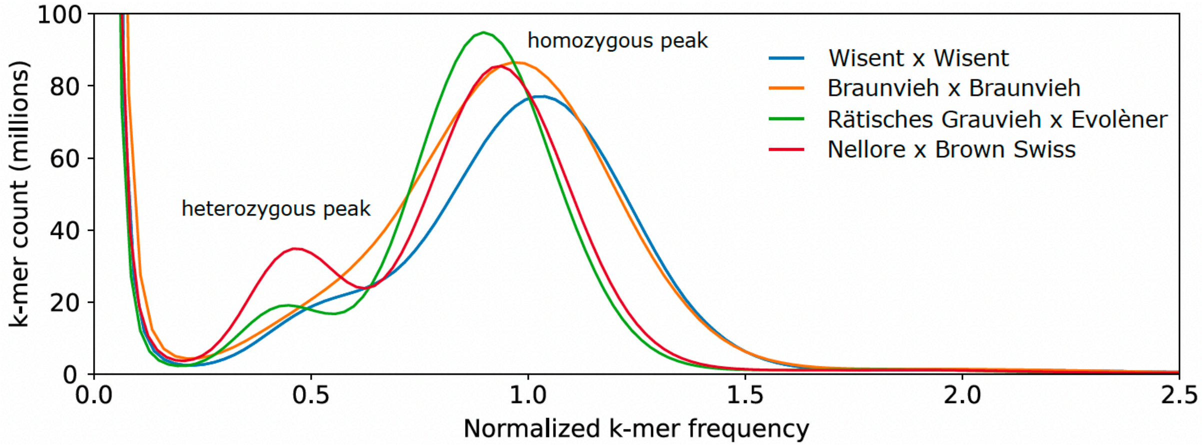

Supplementary Figure 1: Distinct k-mer distribution for four samples. Although the wisent sample has lengthy runs of homozygosity, its heterozygous peak is slightly more defined than in the Braunvieh x Braunvieh sample.

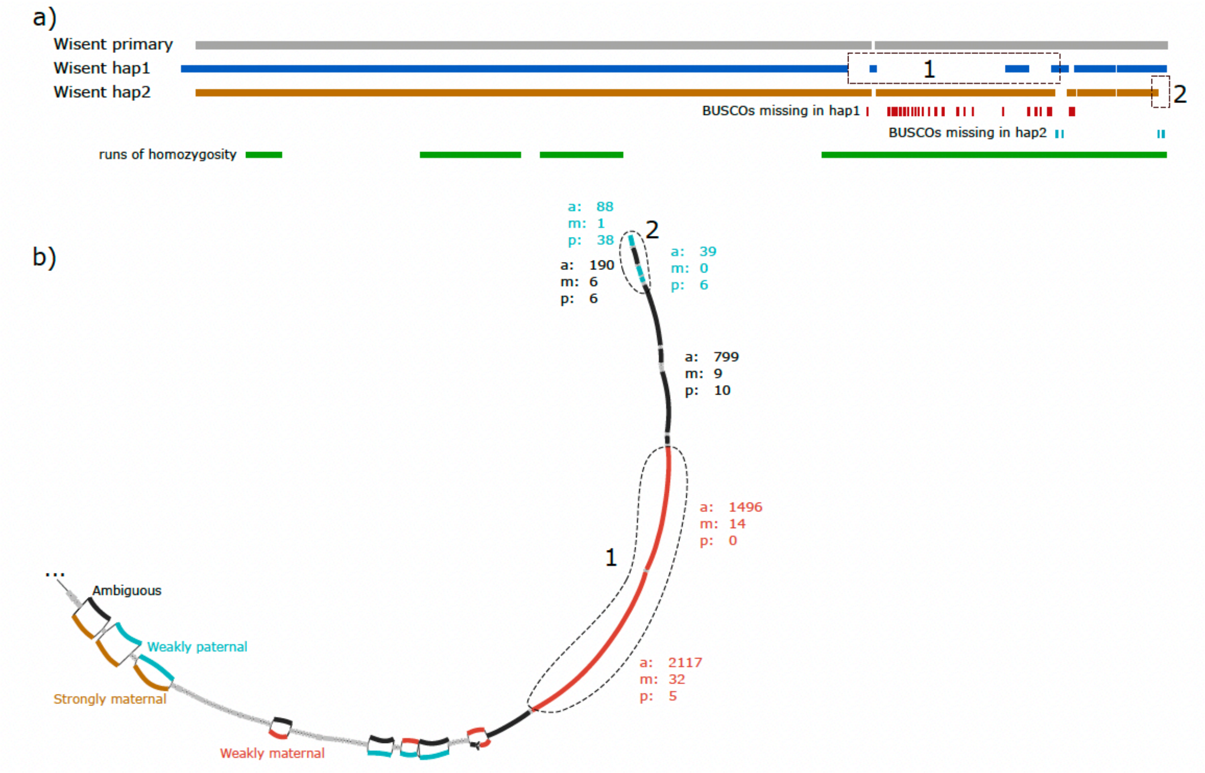

Supplementary Figure 2: Missing BUSCO genes are located in regions entirely missing from one haplotype. (a) Pangenome synteny of the primary and haplotype-resolved wisent assemblies on chromosome 17. Missing BUSCOs are shown below, corresponding to sequence missing from one of the two haplotype-resolved assemblies. (1) is an approximately 15 Mb of sequence containing 24 BUSCOs missing from haplotype 1 but present in haplotype 2 and the primary assembly while (2) is approximately 1 Mb and contains 2 BUSCOs missing from haplotype 2. Runs of homozygosity larger than 2 Mb and fewer than 2 heterozygous variants per 10 Kb are shown. (b) An annotated region of the hifiasm primary unitig graph on chromosome 17 from 40 Mb to the end. Large unitigs can be assigned as Ambiguous (black) if there is no imbalance in parental haplotype tagging. Unitigs could also have primarily maternal tagged reads (brown), primarily ambiguous but biased towards maternal (orange), or primarily ambiguous but biased towards paternal (blue). This region did not contain any strongly paternal tagged unitigs. The major haplotype-resolved errors from regions (1) and (2) were weakly maternal or paternal respectively, located within long runs of homozygosity. The sequences appear to be homozygous but were incorrectly assigned to only one haplotype during haplotype-phasing of this graph.

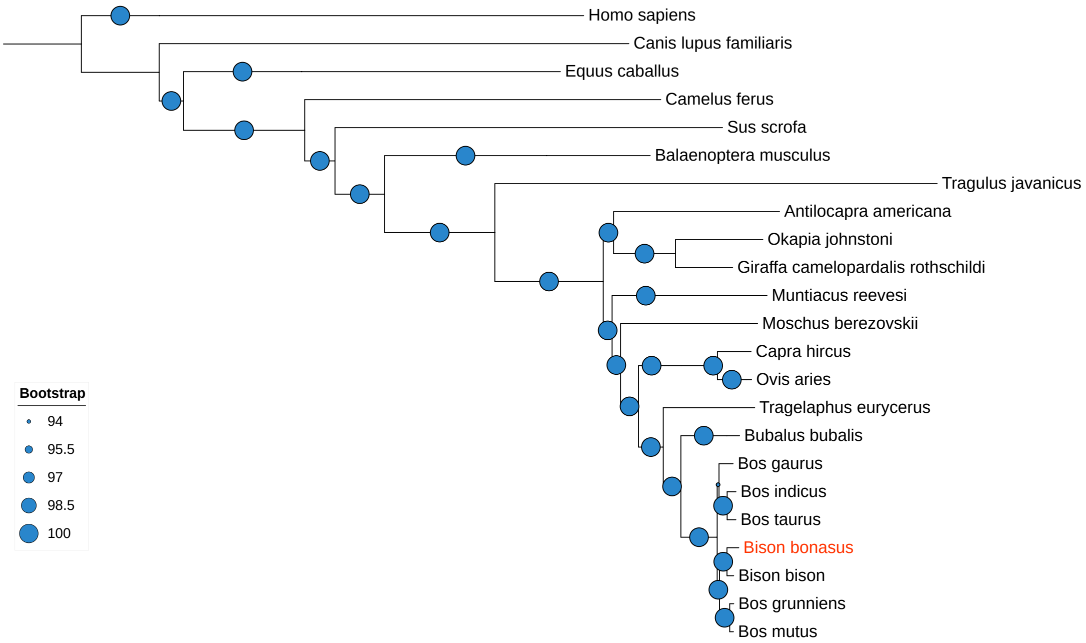

Supplementary Figure 3: Bootstrap confidence intervals of wisent phylogeny. *Bos gaurus* has lower support (94/100) for joining the cattle clade rather than the bison/yak clades compared to all other branchings. The gaur sample considered here may have some level hybridization with cattle or introgression responsible for the more ambiguous placement.

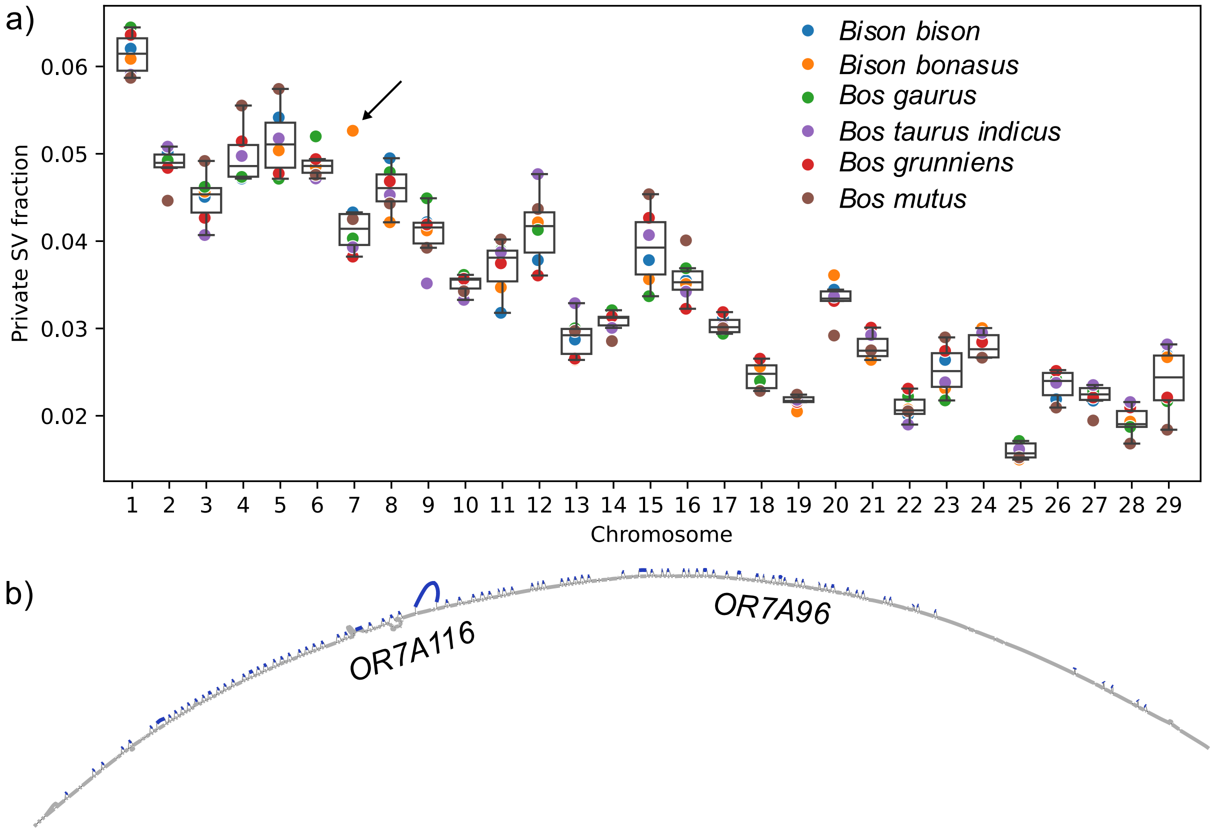

Supplementary Figure 4: Excess of private structural variants to wisent on chromosome 7. (a) Chromosome 7 was a substantial outlier when considering the number of SVs private to wisent compared to the total number of SVs per chromosome and was not a pattern observed in any other sample. (b) Most private SVs to wisent were clustered between 10 and 10.6 Mb on chromosome 7, a region containing 12 annotated protein-coding genes. Two of these genes are explicitly identified as part of the olfactory receptor family 7 subfamily A (OR7A), while many of the remaining genes have orthologous relationships to the OR7A subfamily.

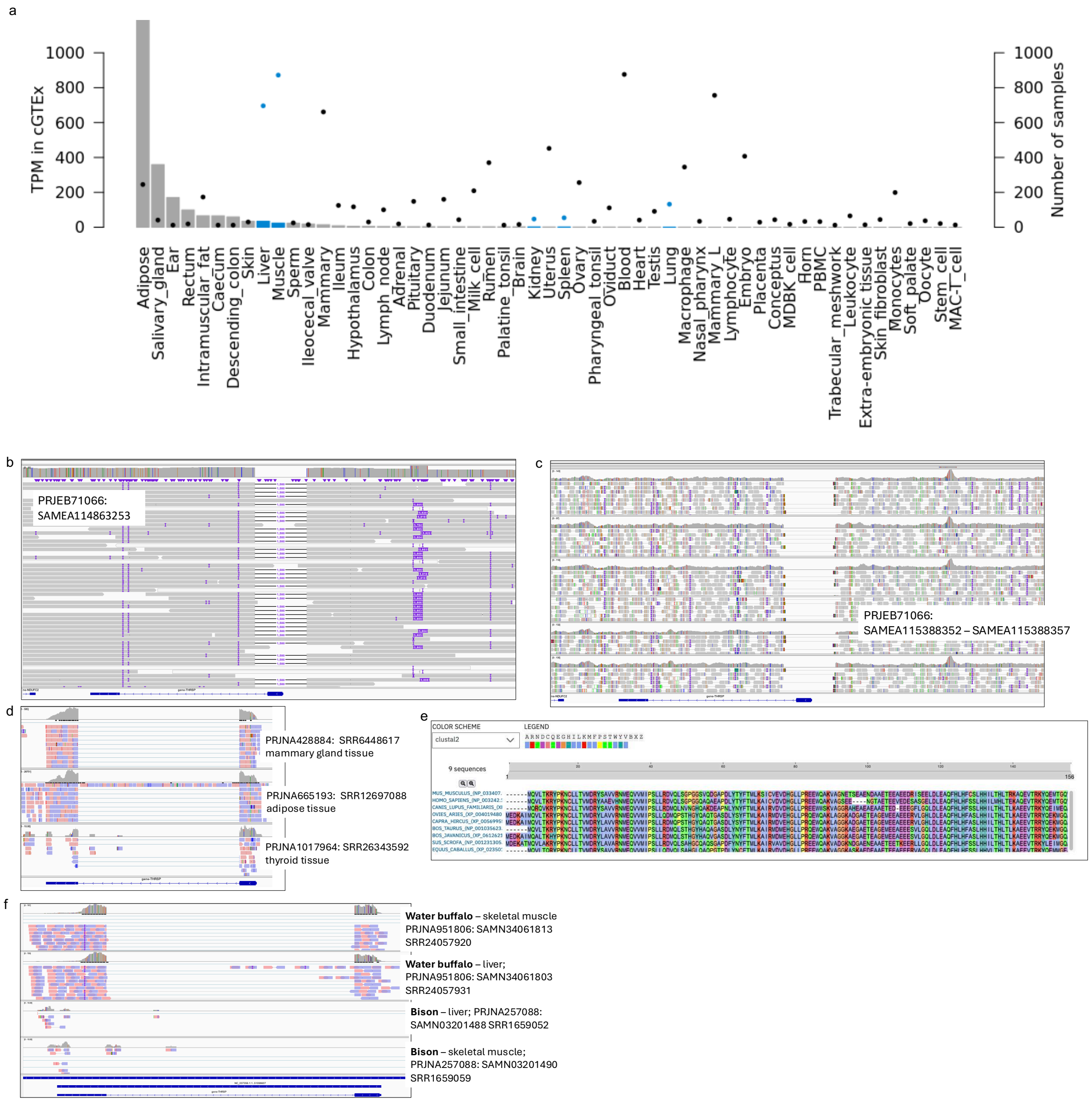

Supplementary Figure 5: Validation of a large deletion uncovered by the pangenome analysis. (a) Expression of *THRSP* in 55 tissues from the cattle GTEx dataset. The bars represent TPM values, and the black dots represent the number of samples per tissue. The y axis is truncated at 1000. Blue colour represents tissues for which transcriptome data are also available for the 3 years old bison cow. (b) Alignment of the wisent F1 HiFi reads against ARS-UCD1.2 confirm deletion of 1,580 bp sequence encompassing the first coding exon of THRSP encoding thyroid hormone responsive protein. (c) Alignment of six short-read sequenced wisent genomes support the deletion. (d) Alignment of RNA sequencing data from three cattle tissues (mammary gland, adipose, thyroid) confirm expression of the first THRSP exon. (e) Multi-species alignment of the THRSP protein sequence. (f) Alignment of RNA sequencing data from skeletal muscle and liver tissue from water buffalo and bison. Both exons are expressed in water buffalo while neither of the exons is expressed in bison.

Supplementary Table 1: Individual ROH statistics based on the method of Bortoluzzi et al. (2020) [1].

Sample name	Heterozygosity	Long ROH*	Sum long ROH (bp)	Sum_ROH_length (bp)	FROH_all	FROH_long_ROHs
Lupita	0.00107935	148	909300000	1405940000	0.52	0.34
Uranda	0.00113541	134	836990000	1368090000	0.51	0.31
Enrik	0.00117748	146	817220000	1334160000	0.50	0.30
Urim	0.00117547	140	775950000	1340300000	0.50	0.29
Urano	0.00117001	139	923210000	1382490000	0.52	0.34
Langnau	0.00095343	168	1047470000	1541170000	0.57	0.39
BBO_3569	0.00078130	132	435750000	1554210000	0.58	0.16
BBO_3574	0.00083465	121	386690000	1514040000	0.56	0.14
American_bison_1	0.00130607	27	96360000	689510000	0.27	0.04
American_bison_2	0.00141251	13	70050000	650140000	0.25	0.03
American_bison_3	0.00134921	13	69400000	657720000	0.26	0.03
American_bison_4	0.00135144	20	85790000	651270000	0.25	0.03
Angus	0.00108068	67	139410000	639190000	0.24	0.05
Hereford	0.00097061	83	190840000	720720000	0.27	0.07
Charolais	0.00104535	85	184530000	692890000	0.26	0.07
Holstein	0.00107978	51	137390000	609440000	0.22	0.05

* Long ROH are defined as regions >2Mb

Supplementary Table 2: Bin-then-assemble approach does not address all haplotype-resolved issues**.**

Haplotype	Approach	Missing BUSCOs	Phase switch	N50	Effective haploid coverage
Paternal	Trio	487	0.0068	74.4	21.9*
Paternal	Binned	285	0.1086	14.4	30.3†
Maternal	Trio	937	0.01029	58.9	21.9*
Maternal	Binned	167	0.2865	14.2	30.0†

* Trio-assembly uses 43.8-fold coverage of the F1
† Approximately 8.5-fold coverage of ambiguous reads are assigned to both haplotypes, so the total coverage exceeds the F1 coverage

Supplementary Table 3: Summary of total repeat content in the wisent (Bison bonasus) primary assembly

Species	*Bison bonasus**	*Bison bison*	*Bison bison**	*Bos taurus*	*Bos taurus**
**Bases masked - bp**	1502142752 (48.90%)	1182179069 (43.52%)	1175212623 (43.27%)	1096723215 (41.73%)	1101218423 bp (41.90 %)
**SINEs**					
Number	937006	1021871	926001	915814	916206
Length occupied - bp	117355500	148491961	115189672	141309445	113245923
Percentage of sequence - %	3.82%	5.47%	4.24%	5.38%	4.31%
**LINEs**					
Number	2465919	2336432	2412734	2298538	2386203
Length occupied - bp	852775379	788429808	821074888	790429168	806393477
Percentage of sequence - %	27.76%	29.03%	30.23%	30.07%	30.68%
**LTR elements**					
Number	430989	384374	383280	395402	371858
Length occupied - bp	136450427	100583169	97075805	97010720	92385468
Percentage of sequence - %	4.44%	3.70%	3.57%	3.69%	3.51%
**Total interspersed repeats**					
Number	-	-	-	-	-
Length occupied - bp	1175862885	1103330778	1096209613	1071344489	1073907749
Percentage of sequence - %	38.28%	40.62%	40.36%	40.76%	40.86%
**Small RNA**					
Number	608646	726966	602228	496000	595068
Length occupied - bp	85027755	115638641	83595795	79845917	82049940
Percentage of sequence - %	2.77%	4.26%	3.08%	3.04%	3.12%
**Satellites**					
Number	193392	7760	12123	1222	2646
Length occupied - bp	297549040	55372732	55013119	1713287 bp	3388650
Percentage of sequence - %	9.69%	2.04%	2.03%	0.07%	0.13%
**Simple repeats**					
Number	525735	487657	497281	493757	499029
Length occupied - bp	21182427	19150255	19669258	19480905	19660788
Percentage of sequence - %	0.69%	0.71%	0.72%	0.74%	0.75%
**Low complexity**					
Number	86734	84987	85846	84283	84626
Length occupied - bp	4270831	4169903	4210080	4141661	4153261
Percentage of sequence - %	0.14%	0.15%	0.16%	0.16%	0.16%
**Unclassified**					
Number	139278	121141	119424	7029	116983
Length occupied - bp	26207508	23888058	22477944	2090001	21856944
Percentage of sequence - %	0.85%	0.88%	0.83%	0.08%	0.83%

*Masked using wisent repeat library

Supplementary Table 4: Samples information of trio binning

Sample	F1 HiFi accession	Sire Illumina accession	Dam Illumina accession
Wisent x Wisent	SAMEA114863253	SAMEA115388356	SAMEA115388355
Braunvieh x Braunvieh	SAMEA115129361	SAMEA115121772	SAMEA115121773
Rätisches Grauvieh x Simmental	SAMEA115129365	SAMEA115121766	SAMEA115121767
Nellore x Brown Swiss	SAMEA7765441	SAMEA9533783	SAMEA6163185

Supplementary Table 5: List of additional species used in the phylogenetic tree construction

Assembly Accession	Organism Name	Assembly Level	Assembly BioProject Accession	Assembly BioSample Accession
GCA_007570785.1	Antilocapra americana	Scaffold	PRJNA526246	SAMN11091591
GCA_963879515.1	Bison bonasus	Chromosome	PRJEB71066	SAMEA114863253
-	Bos gaurus	Chromosome	PRJEB48481	SAMEA10563833
GCA_009493645.1	Bos grunniens	Chromosome	PRJNA551500	SAMN12153487
-	Bos indicus	Chromosome	PRJEB42335	SAMEA7765441
GCA_027580195.1	Bos mutus	Chromosome	PRJNA720245	SAMN18643077
GCA_002263795.4	Bos taurus	Chromosome	PRJNA391427	
GCA_030254855.1	Bison bison	Chromosome	PRJNA945428	SAMN33913332
GCA_019923935.1	Bubalus bubalis	Chromosome	PRJNA525182	SAMN11104991
GCA_001704415.2	Capra hircus	Chromosome	PRJNA290100	SAMN03863711
GCA_017591445.1	Giraffa camelopardalis rothschildi	Chromosome	PRJNA627604	SAMN14677183
GCA_022376915.1	Moschus berezovskii	Scaffold	PRJNA752261	SAMN20589840
GCA_020226045.1	Muntiacus reevesi	Chromosome	PRJNA640966	SAMN15339013
GCA_024291935.2	Okapia johnstoni	Chromosome	PRJNA708170	SAMN18755936
GCA_016772045.1	Ovis aries	Chromosome	PRJNA675594	SAMN17575729
GCA_004024965.2	Tragulus javanicus	Scaffold	PRJNA399456	SAMN07678116
GCA_935064755.1	Tragelaphus eurycerus	Contig	PRJEB50638	SAMEA13301202
GCA_009873245.3	Balaenoptera musculus	Chromosome	PRJNA554522	SAMN12287136
GCA_009834535.1	Camelus ferus	Chromosome	PRJNA550024	SAMN12102236
GCA_000003025.6	Sus scrofa	Chromosome	PRJNA13421	SAMN02953785
GCA_002863925.1	Equus caballus	Chromosome	PRJNA421018	SAMN02953672
GCA_011100685.1	Canis lupus familiaris	Chromosome	PRJNA587469	SAMN13230619
GCA_009914755.4	Homo sapiens	Chromosome	PRJNA559484	SAMN03255769

Supplementary references

1. Bortoluzzi C, Bosse M, Derks MFL, Crooijmans RPMA, Groenen MAM, Megens HJ. The type of bottleneck matters: Insights into the deleterious variation landscape of small managed populations. Evolutionary Applications. 2020;13(2):330–41.
