# Supplementary material for: Genome assembly of wisent (Bison bonasus) uncovers a deletion that likely inactivates the THRSP gene
Source: Commun Biol. 2024 Nov 27;7:1580. doi: 10.1038/s42003-024-07295-y (PMC11603333; doi:10.1038/s42003-024-07295-y)
Supplement: Supplementary file 3 — Description of Additional Supplementary File [file 42003_2024_7295_MOESM3_ESM.pdf]

Description of additional supplementary file

File name: Supplementary Data 1
Description: VCF file with SV annotations

File name: Supplementary Data 2
Description: Sample information and quality assessment of the raw resequencing data

File name: Supplementary Data 3
Description: Accession numbers of the DNA and RNA sequencing data used to investigate the THRSP deletion

File name: Supplementary Data 4
Description: The source data behind the graphs in the paper
