# Supplementary material for: Genome assembly of wisent (Bison bonasus) uncovers a deletion that likely inactivates the THRSP gene
Source: Commun Biol. 2024 Nov 27;7:1580. doi: 10.1038/s42003-024-07295-y (PMC11603333; doi:10.1038/s42003-024-07295-y)
Supplement: Supplementary file 8 — Reporting Summary [file 42003_2024_7295_MOESM8_ESM.pdf]

nature portfolio

Corresponding author(s): Hubert Pausch

Last updated by author(s): Nov 11, 2024

Reporting Summary

Nature Portfolio wishes to improve the reproducibility of the work that we publish. This form provides structure for consistency and transparency in reporting. For further information on Nature Portfolio policies, see our Editorial Policies and the Editorial Policy Checklist.

Statistics

For all statistical analyses, confirm that the following items are present in the figure legend, table legend, main text, or Methods section.

n/a	Confirmed	
☐	☒	The exact sample size (*n*) for each experimental group/condition, given as a discrete number and unit of measurement
☐	☒	A statement on whether measurements were taken from distinct samples or whether the same sample was measured repeatedly
☐	☒	The statistical test(s) used AND whether they are one- or two-sided<br>*Only common tests should be described solely by name; describe more complex techniques in the Methods section.*
☐	☒	A description of all covariates tested
☒	☐	A description of any assumptions or corrections, such as tests of normality and adjustment for multiple comparisons
☐	☒	A full description of the statistical parameters including central tendency (e.g. means) or other basic estimates (e.g. regression coefficient) AND variation (e.g. standard deviation) or associated estimates of uncertainty (e.g. confidence intervals)
☐	☒	For null hypothesis testing, the test statistic (e.g. *F*, *t*, *r*) with confidence intervals, effect sizes, degrees of freedom and *P* value noted<br>*Give P values as exact values whenever suitable.*
☒	☐	For Bayesian analysis, information on the choice of priors and Markov chain Monte Carlo settings
☒	☐	For hierarchical and complex designs, identification of the appropriate level for tests and full reporting of outcomes
☒	☐	Estimates of effect sizes (e.g. Cohen's *d*, Pearson's *r*), indicating how they were calculated

Our web collection on statistics for biologists contains articles on many of the points above.

Software and code

Policy information about availability of computer code

Data collection	We used PacBio and Illumina sequencing to generate genome data for wisent samples. This is described in full detail in material and methods.<br>HiFi reads of the F1 are available in the ENA at the study accession PRJEB71066 under sample accession SAMEA114863253. Illumina paired-end reads of six captive wisents are available in the ENA at the study accession PRJEB71066 under sample accessions SAMEA115388352, SAMEA115388353, SAMEA115388354 (F1), SAMEA115388355 (dam), SAMEA115388356 (sire), SAMEA115388357.<br>We used publicly available DNA and RNA sequencing data from wisent, bison, cattle, and buffalo samples. Accession numbers of these data are provided in Supplementary Tables 1, 2, and 7.<br>We used publicly available assemblies for eleven cetartiodactyla species for which the Genbank accession numbers are provided in Supplementary Table 3.<br>We used a publicly available TPM matrix (https://zenodo.org/records/7560235) built by the cattle Genotype-Tissue Expression (GTEx) project
Data analysis	The following publicly available software tools were used; all software is cited in the material and methods section.<br>hifiasm v0.19.5 (Cheng et al., 2021)<br>yak v0.1 (https://github.com/lh3/yak)<br>RagTag v2.1.0 (Alonge et al., 2022)<br>merqury (6b5405) (Rhie et al., 2020)<br>meryl (https://github.com/marbl/meryl)<br>calN50.js (https://github.com/lh3/calN50)<br>compleasm v0.2 (Huang and Li, 2023)<br>bandageNG v2022.9 (Wick et al., 2015)<br>canu v2.2 (Koren et al., 2017)<br>minimap2 v2.26 (Li, 2018)

SAMtools v1.19.2 (Li et al., 2009)
RepeatModeler v2.0.4 (Flynn et al., 2020)
RECON v1.08 (Bao and Eddy, 2002)
RepeatScout v1.0.6 (Price et al., 2005)
Tandem Repeats Finder v4.09.1 (Benson, 1999)
RepeatMasker v4.1.5 (https://www.repeatmasker.org/).
MAFFT v7.490 (Katoh and Standley, 2013)
trimAl v1.4 (Capella-Gutiérrez et al., 2009)
RaxML v8.2.12 (Stamatakis, 2014)
Chromosomal Orthologous Link (https://github.com/chulbioinfo/chrorthlink)
genoPlotR library (Guy et al., 2010)
liftoff v1.6.3 (Shumate and Salzberg, 2021)
minigraph v0.20 (Li et al., 2020)
mash v2.3 (Ondov et al., 2016).
vg v1.55.0 (Liao et al., 2023)
BCFtools v1.19 (Danecek et al., 2021)
upsetplot v0.9 (https://github.com/jnothman/UpSetPlot).
SciPy v1.12 (Virtanen et al., 2020)
Variant Effect Predictor (VEP) (McLaren et al., 2016)
FastQC v0.11.9 (https://www.bioinformatics.babraham.ac.uk/projects/fastqc/)
BWA v0.7.17-r1188 (Li, 2013)
samblaster v0.1.24 (Faust and Hall, 2014)
sambamba v0.8.1 (Tarasov et al., 2015)
bamtools v2.5.1 (Barnett et al., 2011)
Qualimap v2.3 (Okonechnikov et al., 2016)
Freebayes v0.9.21 (Garrison and Marth, 2012)
STAR v2.7.9a (Dobin et al., 2013)
Integrative Genomics Viewer v2.14.0 (Robinson et al., 2011)
kallisto v0.46.1 software (Bray et al., 2016)
r8s (https://github.com/iTaxoTools/pyr8s)
FigTree v1.4.4 (https://github.com/rambaut/figtree)
Codes used in this study are available in GitHub: https://github.com/cbortoluzzi/WisentGenomeAssemblyand at zenodo (https://zenodo.org/records/14056475). A Code availability statement is included in the manuscript.

For manuscripts utilizing custom algorithms or software that are central to the research but not yet described in published literature, software must be made available to editors and reviewers. We strongly encourage code deposition in a community repository (e.g. GitHub). See the Nature Portfolio guidelines for submitting code & software for further information.

Data

Policy information about availability of data

All manuscripts must include a data availability statement. This statement should provide the following information, where applicable:

- Accession codes, unique identifiers, or web links for publicly available datasets
- A description of any restrictions on data availability
- For clinical datasets or third party data, please ensure that the statement adheres to our policy

The primary assembly of the wisent is publicly available in the European Nucleotide Archive (ENA) under accession GCA_963879515.1 (https://www.ebi.ac.uk/ena/browser/view/GCA_963879515.1). The annotation of the primary assembly is currently underway at Ensembl. HiFi reads of the F1 are available in the ENA at the study accession PRJEB71066 under sample accession SAMEA114863253. Illumina paired-end reads of six captive wisents are available in the ENA at the study accession PRJEB71066 under sample accessions SAMEA115388352, SAMEA115388353, SAMEA115388354 (F1), SAMEA115388355 (dam), SAMEA115388356 (sire), SAMEA115388357. The source data behind the graphs in the paper can be found in Supplementary Data 4.

Research involving human participants, their data, or biological material

Policy information about studies with human participants or human data. See also policy information about sex, gender (identity/presentation), and sexual orientation and race, ethnicity and racism.

Reporting on sex and gender	NA
Reporting on race, ethnicity, or other socially relevant groupings	NA
Population characteristics	NA
Recruitment	NA
Ethics oversight	NA

Note that full information on the approval of the study protocol must also be provided in the manuscript.

Field-specific reporting

Please select the one below that is the best fit for your research. If you are not sure, read the appropriate sections before making your selection.

☒ Life sciences ☐ Behavioural & social sciences ☐ Ecological, evolutionary & environmental sciences

For a reference copy of the document with all sections, see nature.com/documents/nr-reporting-summary-flat.pdf

Life sciences study design

All studies must disclose on these points even when the disclosure is negative.

Sample size	We sequenced one trio for trio-binning (parents and offspring).<br>No specific calculations were made to pre-determine sample size.
Data exclusions	we applied standard quality control with FastQC to exclude sequencing reads with low quality
Replication	NA
Randomization	NA
Blinding	NA

Reporting for specific materials, systems and methods

We require information from authors about some types of materials, experimental systems and methods used in many studies. Here, indicate whether each material, system or method listed is relevant to your study. If you are not sure if a list item applies to your research, read the appropriate section before selecting a response.

Materials & experimental systems

n/a	Involved in the study	
☒	☐	Antibodies
☒	☐	Eukaryotic cell lines
☒	☐	Palaeontology and archaeology
☐	☒	Animals and other organisms
☒	☐	Clinical data
☒	☐	Dual use research of concern
☒	☐	Plants

Methods

n/a	Involved in the study	
☒	☐	ChIP-seq
☒	☐	Flow cytometry
☒	☐	MRI-based neuroimaging

Animals and other research organisms

Policy information about studies involving animals; ARRIVE guidelines recommended for reporting animal research, and Sex and Gender in Research

Laboratory animals	*For laboratory animals, report species, strain and age OR state that the study did not involve laboratory animals.*
Wild animals	We obtained blood and tissue samples of six captive wisents (two female, four males) from the Bern animal park and Langenberg animal park in Switzerland.
Reporting on sex	NA
Field-collected samples	NA
Ethics oversight	No animals were sampled for this study. No ethics approval was required for this study.

Note that full information on the approval of the study protocol must also be provided in the manuscript.

Plants

Seed stocks	NA
Novel plant genotypes	NA
Authentication	NA
